# A new hope for patients with Huntington's disease?

**DOI:** 10.1016/j.eclinm.2025.103612

**Published:** 2025-10-23

**Authors:** eClinicalMedicine

On Sept 24, a gene therapy company uniQure released preliminary positive data from its pivotal phase 1/2 trial of AMT-130, a novel treatment for Huntington's disease. The announcement sparked widespread discussion in the medical community and renewed hope among patients with Huntington's disease and clinicians. Huntington's disease so far has been thought to be an incurable disease with little recent progress in the development of new treatment modalities.

Huntington's disease is a hereditary neurodegenerative disorder caused by the mutation of the *HTT* gene, which encodes the protein Huntingtin, with an abnormal expansion of over 40 repeats of the trinucleotide CAG. It is expressed in many tissues, but the highest expression levels are within the brain. The exact molecular role of the protein is not yet fully understood, but it is involved in the survival and development of neurons, axonal transport, and apoptosis. The mutated form of Huntingtin accumulates in neurons and leads to their dysfunction and death.

Patients with Huntington's disease usually develop symptoms when they are in their 30s or 40s. When Huntington's disease appears before the age of 20 years, it is referred to as juvenile Huntington's disease. Symptoms can vary vastly from patient to patient but are mostly related to motor, cognitive, and psychiatric symptoms. Impairment of body movements usually manifest in involuntary movements or trouble speaking and swallowing. Huntington's disease can also affect mental health, causing difficulties with thinking and planning and can lead to personality changes. Overall, Huntington's disease is a progressive disease with no cure or treatment options available. The average life expectancy after symptom onset is 10–20 years with a steep decline in the quality of life, often requiring full-time care in later stages. Although new treatment options for Huntington's disease are urgently needed, the recent AMT-130 gene therapy trial offers a promising source of hope. Current treatment options focus primarily on symptom management and supportive measures only.

AMT-130 is a one-time gene therapy targeting mutant *HTT*. It uses an adeno-associated virus (AAV5) to deliver a microRNA directly into the striatum (a brain region heavily affected by Huntington's disease). The microRNA is intended to silence *HTT*, reducing production of the toxic huntingtin protein that damages the neurons. The therapy itself is administered via MRI-guided brain surgery, requiring a 12–20-h procedure.

This early phase 1/2 trial involved 29 patients with early-stage Huntington's disease, 17 of whom received high-dose AMT-130 and 12 patients who received the low dose. Results from the prespecified primary endpoint, disease progression as measured by the composite Unified Huntington's Disease Rating Scale (cUHDRS) at 36 months compared with a propensity score-matched external control, were reported in a Press Release by the developing company on Sept 24, 2025. Outcomes are available for 12 patients per dose group. Patients in the high-dose group showed a significant 75% reduction in disease progression measured as the primary endpoint. Patients in this group also showed improvements in motor and cognitive function. Furthermore, cerebrospinal neurofilament light protein (NfL) levels as an independent read-out decreased by 8.2%, suggesting reduced neurodegeneration. NfL levels are considered to be a biomarker for neuronal damage. Similarly, elevation of NfL in the cerebrospinal fluid has been shown to be strongly associated with greater clinical severity of Huntington's disease.

Although the drug is not yet approved by the US Food and Drug Administration (FDA) or the European Medicines Agency, the developing company plans to submit an application to the FDA in early 2026. So far, AMT-130 has received breakthrough therapy, orphan, and fast track designation. With this designation, the way is paved to expedite the development for AMT-130 to become available to more patients with Huntington's disease in follow-up trials.

Taking all the good news together, this is certainly a scientific breakthrough and there are a lot of reasons to be optimistic. As for all early trial results, and all excitement and optimism aside, we need to acknowledge that the data are very preliminary with only 12 patients analysed in each group. The study and the full data set first need to undergo rigorous peer review to ensure publication of all the data in an unbiased manner. Looking into the trial registration, there are certainly some issues that need consideration regarding the many changes made to the registration throughout the trial. Larger and longer-term randomised controlled trials are needed to confirm the true efficacy, safety, and optimal dose of the drug. For now, patients with Huntington's disease can have genuine hope for a potential treatment option for this progressive and ultimately fatal condition.

